# Endothelial nitric oxide synthase gene Glu298Asp polymorphism in patients with coronary artery disease

**DOI:** 10.4103/0256-4947.59370

**Published:** 2010

**Authors:** Saeedeh Salimi, Mohsen Firoozrai, Hamid Zand, Alireza Nakhaee, Sayed M. Shafiee, Heidar Tavilani, Ahmad Mohebbi

**Affiliations:** a From the Cellular and Molecular Research Center, School of Medicine, Zahedan University of Medical Sciences, Zahedan, Iran; b From the Department of Biochemistry, School of Medicine, Zahedan University of Medical Sciences, Zahedan, Iran; c From the Department of Biochemistry, School of Medicine, Iran University of Medical Sciences, Tehran, Iran; d From the Department of Nutrition, School of Medicine, Shahid Beheshty University of Medical Sciences, Tehran, Iran; e From the Department of Biochemistry, Medical School, Hamadan University of Medical Sciences, Hamadan, Iran; f From the Department of Cardiology, Shahid Rajaee Heart Hospital, Iran University of Medical Sciences, Tehran, Iran

## Abstract

**BACKGROUND AND OBJECTIVES:**

Endo-derived nitric oxide (NO) is synthesized from L-arginine by endothelial nitric oxide synthase (NOS3). Since reduced NO synthesis in endothelial cells has been implicated in the development of coronary atherosclerosis, we investigated the association of NOS3 gene polymorphisms and coronary artery disease (CAD) in an Iranian population.

**SUBJECTS AND METHODS:**

We studied the NOS3 gene Glu298Asp polymorphism in 241 CAD patients with positive coronary angiograms (i.e., >50% stenosis affecting at least one coronary vessel) in Shahid Rajaee Heart Hospital and 261 control subjects without a history of symptomatic CAD. The NOS3 gene polymorphism was analyzed by polymerase chain reaction and restriction fragment length polymorphism. Lipid profile and other risk factors were also determined.

**RESULTS:**

The genotype frequencies of Glu298Asp polymorphism for Glu/Glu, Glu/Asp, and Asp/Asp were 61.3%, 32.2%, and 6.5%, respectively, in control subjects, and 46.5%, 42.7%, and 10.8% in CAD patients, respectively. The genotype frequencies differed significantly between the two groups (*P*=.003). The frequencies of the Asp alleles were 32.2% and 22.6% for CAD patients and control subjects, respectively; the difference between the two groups was statistically significant (*P*=.001; odds ratio=1.6). Plasma lipids, except HDL-C, were also significantly increased in the CAD groups.

**CONCLUSION:**

These results suggest that CAD is associated with Glu298Asp polymorphism of the NOS3 gene in our population and that this polymorphism is an independent risk factor for CAD.

Nitric oxide (NO) is synthesized from L-arginine and molecular oxygen by a family of three enzymes: NOS1, NOS2, and NOS3.^1^,^2^ NO is the most powerful endogenous vasodilator known. It can also inhibit the adhesion, aggregation, and recruitment of platelets; inhibit vascular smooth muscle cell migration and growth; regulate some vessel–platelet interactions; and limit the oxidation of atherogenic low-density lipoproteins.^1^ These actions are all mediated by the activation of soluble guanylate cyclase and the consequent increase in the concentration of cyclic GMP in target cells.^3^

The constitutive endothelial nitric oxide synthase (NOS3) is expressed in the endothelium and is encoded by a 26-exon gene located on chromosome 7q35 to 36; the gene, with a total size of 21 kb, encodes an mRNA of 4052 nucleotides.^4^,^5^ The NOS3 gene is expressionally and functionally regulated through multiple regulatory steps and entails several polymorphisms, some of which bear functional consequences.^4^

Reduction in basal NO release is known to predispose humans to atherosclerosis, hypertension, thrombosis, and vasospasm. On the other hand, high circulating NO concentrations, which occur with excessive inducible NOS expression under pathological conditions, are generally toxic.^6^ Emerging evidence suggests that coronary artery disease (CAD) is related to defects in the generation or function of NO. Markedly increased NO concentrations are reported to be associated with endotoxic shock and exaggerated inflammatory reactions, which may lead to acute hepatic dysfunction and predispose humans to asthma and cardiomyopathy.^6^,^7^

In general, NOS3 gene polymorphisms have been reported to act as ‘susceptibility genes’ in various cardiovascular and pulmonary diseases.^7^ GT substitution in exon 7 of codon 298,^8^ 4a/b polymorphism in intron 4,^9^,^10^ T-786C mutation in the 5'-flanking region,^11^,^12^ and high numbers of CA repeat in intron 13 of the NOS3 gene are also associated with an excess risk for CAD.^13^ Among them, a common variant located in exon 7 (G984T) of the NOS3 gene that modifies its coding sequence (Glu298Asp) has been linked by several groups to the risk for CAD and acute myocardial infarction (MI). On the other hand, other studies failed to show any relationship between the Asp variant and the risk for atherosclerosis.^14^ In the present study, we investigated the associations between the occurrence and severity of angiographically defined CAD and the Glu298Asp polymorphism.

## SUBJECTS AND METHODS

Patients recruited in this study (n=241; 174 males and 67 females) were those who had undergone coronary angiography because of recent MI or angina. They were selected at Tehran Shahid Rajaee Heart Hospital after being angiographically identified (>50% stenosis affecting at least one coronary vessel) as having CAD. The control group consisted of 261 individuals (187 males and 74 females) within the same age range as the patients; they were selected at the same hospital after screening to eliminate individuals with a history of chest pain, diabetes, hypertension, or any other specific illness. Clinical details of these groups are shown in Table 1. All participants were interviewed and data on smoking habits, blood pressure, lipid profile, and any family history of CAD were recorded. The study protocol was approved by the Ethics Committee of Iran University of Medical Sciences and written informed consent was taken from all patients and healthy individuals.

**Table 1:** Clinical characteristics of the studied population.

	CAD patients	Controls	*P* value
No. of subjects	241	261	
Age (years)	53.32 (9.4)	51.8 (8.9)	NS
BMI (kg/m^2^)	27.1 (6.1)	27.5 (19.9)	NS
Smoking n (%)	88 (36.5)	66 (25.3)	.004
Family history n (%)	92 (38)	68 (26)	.002
DBP (mm Hg)	85.4 (13.6)	76 (6.7)	.001
SBP (mm Hg)	129.9 (24.3)	117.5 (139)	.001
Cholesterol (mg/dl)	203.8 (45.6)	188.1 (32.3)	.001
TG (mg/dl)	204 (110.5)	169.5 (87.7)	.001
LDL-C (mg/dl)	116.9 (37.2)	105.6 (33.7)	.001
HDL-C (mg/dl)	46.9 (10.4)	48.3 (10.8)	NS
LDL-C/HDL-C	2.63 (0.93)	2.34 (0.88)	.006

Values are mean (SD); BMI=body mass index; SBP=systolic blood pressure; DBP=diastolic blood pressure; TG=triglyceride; LDL-C=LDL cholesterol; HDL-C=HDL cholesterol; NS=non-significant.

Blood samples were obtained from all subjects after 12 hours of fasting and placed in EDTA tubes and stored at −70°C until the time of assay. Serum concentrations of triglyceride (TG), total cholesterol, low-density lipoprotein (LDL-C), and high-density lipoprotein (HDL-C) were measured by standard methods (ZiestChem Diagnostics, Iran) in the clinical laboratory of the hospital. Genomic DNA was tracted from peripheral blood leukocytes by a standard method (Roche Blood Kit, Germany). Two oligonucleotide primers (sense) 5'-GAC CCT GGA GAT GAA GGC AGG AGA-3'and (antisense) 5'-ACC TCC AGG ATG TTG TAG CGG TGA-3' based on the flanking sequences of the exon 7 in the NOS3 gene were used to amplify the corresponding DNA fragment by the polymerase chain reaction (PCR).^15^ The reaction was performed in a 25-μl final volume and contained 25 pmol of each primer, 0.2 mmol of each deoxynucleoside triphosphate (Roche, Germany),1.5 U Taq DNA polymerase (Fermentas, Lithuania), 50 mmol/l KCL, 2.5 mmol/l MgCl_2_, 10 mmol/l Tris-Hcl (pH=8.3), and 400 ng of genomic DNA. The reaction was done according to the following protocol: initial denaturation at 94°C for 5 min, 30 cycles of denaturation at 94°C for 30 s, annealing at 61°C for 30 s, extension at 72°C for 30 s, and final extension at 72°C for 6 min. The 517-bp PCR fragments were digested with *BanII* restriction enzyme for 16 hours at 37°C. The wild-type allele (G) has no *BanII* cleavage site, whereas the PCR product was cleaved into two fragments of 346 and 171 bp in the presence of the T984.

All statistical analysis was performed with SPSS v. 11.5. Numeric data are presented as mean (standard deviation). The differences between groups were examined by the chi-square test or an independent *t* test as appropriate. ANOVA was used for comparisons among the three groups. Allele frequencies were estimated by the gene-counting method. The frequencies of the alleles and genotypes were compared between patient and control groups by the chi-square test when appropriate. The odds ratio (OR) and 95% confidence intervals (CI) were also estimated. The chi-square test was used to examine the deviation of genotype distribution from the Hardy-Weinberg equilibrium. Logistic regression analysis was used to assess the independent effect of each risk factor in CAD.

## RESULTS

The clinical characteristics of the population are shown in Table 1. Control subjects were matched to the case patients by gender and age. Although BMI and HDL-C showed no significant differences between the patients and the control subjects, systolic blood pressure (SBP) and diastolic blood pressure (DBP), triglyceride, total cholesterol, LDL-C, and LDL-C/HDL-C in patients were significantly higher. The frequency of cigarette smoking and family history of CAD in patients were higher than in controls.

The genotype frequencies of Glu298Asp polymorphism in control subjects were 61.3% for Glu/Glu, 32.2% for Glu/Asp, and 6.5% for Asp/Asp. On the other hand, in CAD patients, the genotype frequencies were 46.5% for Glu/Glu, 42.7% for Glu/Asp, and 10.8% for Asp/Asp (Table 2). Although the distribution of genotypes in both the CAD and the control groups satisfied the Hardy-Weinberg equilibrium, the G894T polymorphism of the NOS3 gene was significantly associated with the presence of CAD in our patients (χ^2^=11.5, *P*=.003).

**Table 2:** Genotype and allele frequencies of Glu298Asp polymorphism of the eNOS gene in CAD patients and controls.

	CAD patients	Controls	Odds ratio (95% CI)	*P* value	χ^2^
Glu298 Asp polymorphism				.003	11.5
Glu/Glu, n (%)	112 (46.5)	160 (61.3)			
Glu/Asp, n (%)	103 (42.7)	84 (32.2)			
Asp/Asp, n (%)	26 (10.8)	17 (6.5)			
**Total**	**241**	**261**	**1.83 (1.28-2.6)**	**.001**	**111.1**

Glu/Glu, n (%)	112 (46.5)	160 (61.3)			
Glu/Asp+Asp/Asp, n (%)	129 (53.5)	101 (38.7)			
**Total**	**241**	**261**	**1.6 (1.23-2.2)**	**.0001**	**11.6**

allele					
Glu (%)	67.8	77.4			
Asp(%)	32.2	22.6			

**Total**	**100**	**100**			

The frequencies of the Glu and Asp alleles were 32.2% and 67.8% for CAD patients and 22.6% and 77.4% for the control subjects, respectively; the differences between the groups were statistically significant (χ^2^=11.6, *P*=.001, odds ratio=1.6). We analyzed the frequencies of Glu/Asp and Asp/Asp genotypes in CAD patients and control subjects according to age (age≤55 years and age >55 years) and found that the frequencies of Glu/Asp and Asp/Asp genotypes were significantly higher in patients of age≤55 years(χ^2^=30.3, *P*=.000, odds ratio=3.84) (Table 3). The logistic regression analysis revealed that the NOS3 mutant allele, smoking, family history, DBP, and LDL-C were independent risk factors for CAD (*P*=.004, *P*=.001, *P*=.006, *P*=.001, and *P*=.001, respectively), whereas DBP, cholesterol, HDL-C, and TG were not independent risk factors for CAD (Table 4).

**Table 3:** Genotype frequencies of Glu298Asp polymorphism of the eNOA gene in CAD patients and controls in different ages.

	CAD patients	Controls	Odds ratio (95% CI)	P value	χ^2^
Glu298Asp polymorphism					
Age≤55 years			3.84 (2.4-6.3)	.001	30.3
Glu/Glu, n (%)	36 (28)	104 (60)		
Glu/Asp+Asp/Asp, n (%)	93 (72)	70 (40)			
**Total**	**129**	**174**			

Age>55 years			0.86 (0.47-1.56)	.6	0.27
Glu/Glu, n (%)	76 (68)	56 (64)			
Glu/Asp+Asp/Asp, n (%)	36 (32)	31 (36)			

**Total**	**112**	**87**			

**Table 4:** Multiple logistic regression analysis with forward stepwise selection (Wald).

Variable	B	SE	Wald	df	Significance	Exp (B)
eNOS mutant allele	0.61	0.21	8.3	1	.004	1.84
Cigarrette smoking	0.85	0.23	13.4	1	.001	2.34
Family history	0.64	0.23	7.5	1	.006	1.9
Diastolic blood pressure	0.1	0.012	69.2	1	.001	1.1
LDL-C	0.12	0.003	15.9	1	.001	1.01
Constant	9.7	1.05	84.3	1	.001	0.000

## DISCUSSION

In addition to established risk factors, genetic risk factors may have important roles in the pathogenesis of coronary atherosclerosis and acute MI. Using the approach of epidemiological studies, it is possible to identify weak susceptible genes in polygenic diseases like coronary heart disease.^16^ In the last decade, the potential link between an increasing number of gene variants and coronary heart disease has been analyzed by several investigators. Due to the protective roles of NO against important events during atherogenesis, the NOS3 gene has been identified as also having other roles in deciding susceptibility to coronary heart disease.^17^,^18^

There are many polymorphisms of the NOS3 gene that have been investigated in relation to CAD. Yashimura et al described a point mutation of guanine to thymine at nucleotide 1998 in exon 7 of the NOS3 gene that resulted in replacement of glutamic acid by aspartic acid at codon 298.^8^ Later investigations also described other polymorphisms in the 5' flanking and some introns of the NOS3 gene. Other studies analyzed the relation of the NOS3 gene variation with the risk of CAD. In an Australian population, Cai et al did not detect any association between NOS3 gene polymorphism and the risk of CAD.^19^ Hibi et al showed that patients who are homozygous for the Glu298Asp polymorphism might be genetically predisposed to acute MI; however, this mutation apparently is not related to the severity of coronary atherosclerosis.^15^ In contrast, Hingorani et al observed an excess of homozygotes for Asp298 variant among patients with CAD. In comparison to Glu298 homozygotes, homozygosity for Asp298 was associated with an odds ratio of 4.2 (95% CI: 2.3 to 7.9).^10^ Lembo et al also detected the association of Glu298Asp polymorphism with the risk of CAD in Italy.^20^ Gardemann showed that younger T allele carriers of the NOS3 Glu298Asp gene polymorphism with various coronary high-risk profiles had an increased risk of CAD and/or MI.^21^ In some of the other studies, such as that by Nassar et al in Canada,^22^ Schomoelzer et al in Australia,^23^ and Jaramillo et al in Chile,^24^ no associations were found between Glu298Asp polymorphism and the risk of CAD. In contrast, Colombo et al,^14^ Kerkeni et al,^25^ Cam et al,^26^ and Berdeli et al^27^ detected a link between this polymorphism and CAD in Italian, Tunisian, and Turkish populations. In our previous study, we found that 4a/b polymorphism was not an independent risk factor for CAD in our population.^28^ In the present study, we have shown that Glu298Asp polymorphism is an independent risk factor for CAD in this Iranian population, especially in younger patients (age≤55 years).

The frequency of the Asp allele was approximately 0.23 in our population, which is very similar to that previously reported in a Tunisian population (0.22);^25^ and slightly higher than that observed in Turkish (0.17)^27^ and Indian (0.15)^29^ populations and lower than that in the British (0.31),^10^ Italian (0.30),^14^ and Canadian populations (0.43).^22^

In conclusion, our results show that genotype frequencies for Glu/Glu, Asp/Glu, and Asp/Asp, and also Asp allele frequency, are significantly different between individuals with and without CAD. We have also shown that the Asp allele of NOS3 Glu298Asp polymorphism and DBP are independent risk factors for CAD. In our study, the sample size was small thus; a larger number of patients and controls needs to be examined to confirm the association between this polymorphism and CAD.
